# Supplementary figures and images for: Efflux pump activation confers mupirocin resistance and enhances rhizosphere fitness in Pseudomonas
Source: Appl Environ Microbiol. 2026 Apr 22;92(5):e02575-25. doi: 10.1128/aem.02575-25 (PMC13188873; doi:10.1128/aem.02575-25)

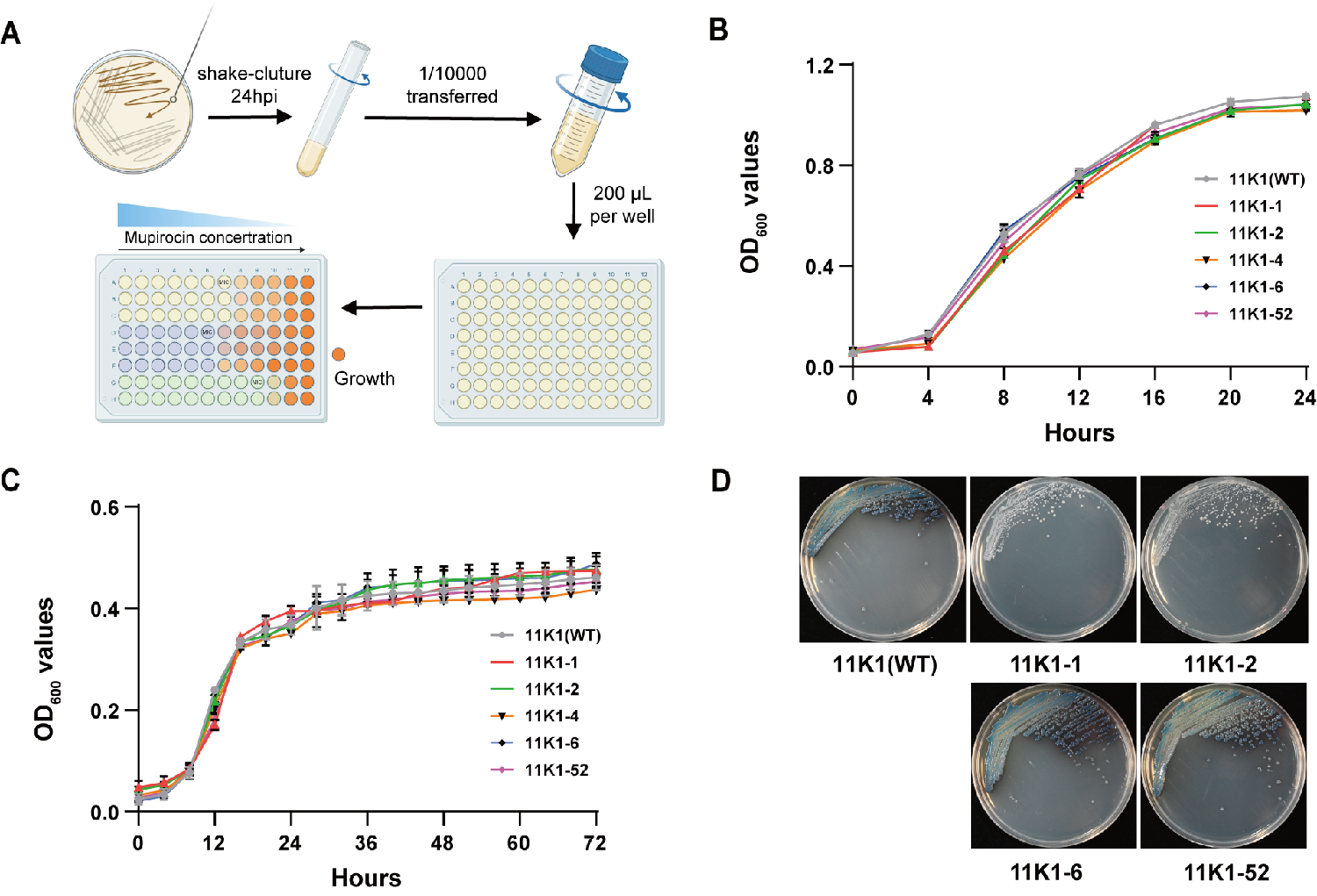

Supplement: Figure S1 — Experimental design of mupirocin MIC assays and biological characteristics of strain 11K1 and its mupirocin-resistant mutants. [file aem.02575-25-s0001.tiff]

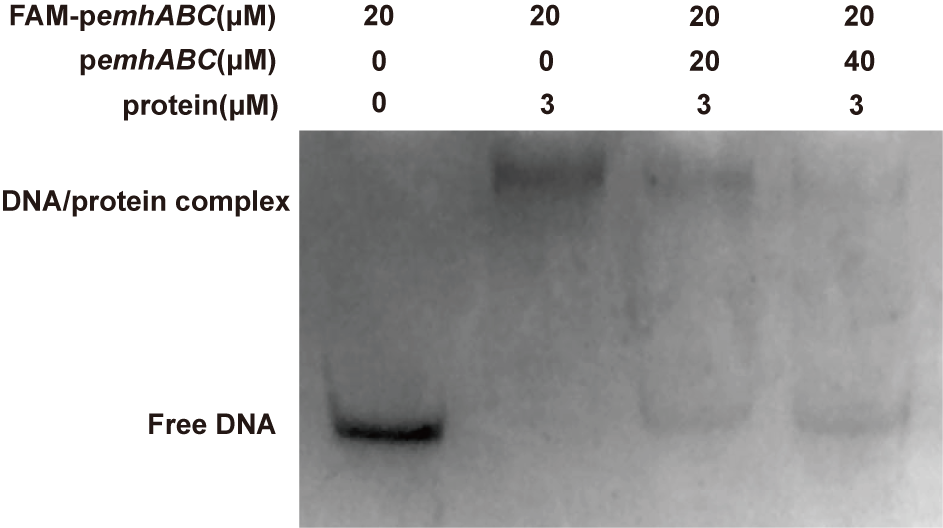

Supplement: Figure S2 — Specificity of EmhR binding to the promoter pemhABC. [file aem.02575-25-s0002.tif]

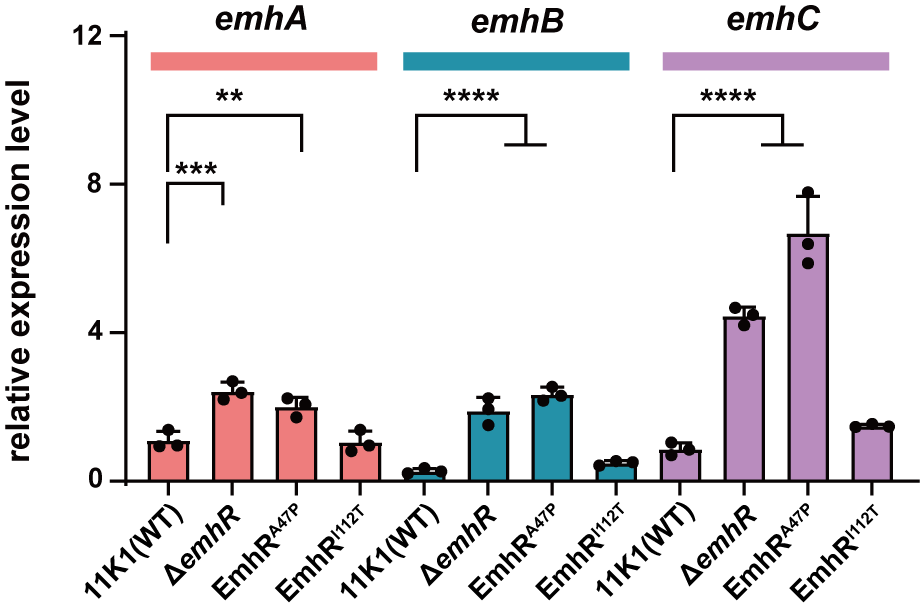

Supplement: Figure S3 — Transcriptional regulation of emhABC by EmhR deletion and point mutations. [file aem.02575-25-s0003.tif]

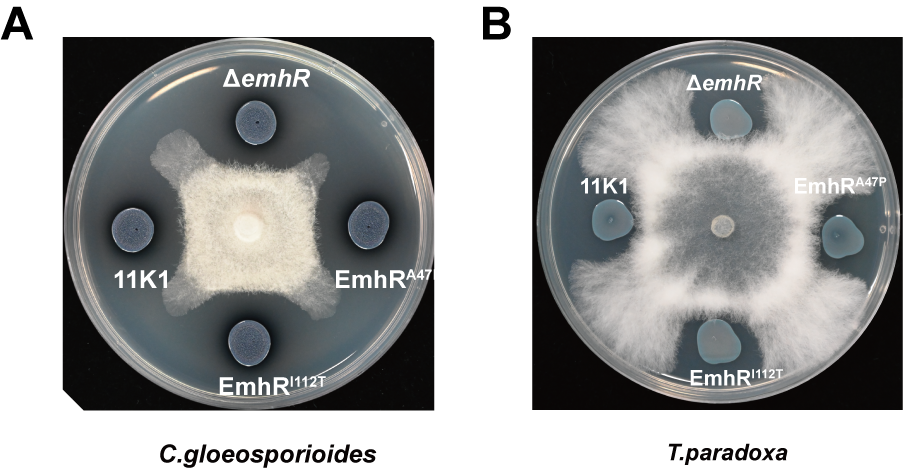

Supplement: Figure S4 — Plate confrontation assays of the ΔemhR mutant and EmhR point mutants against the fungal pathogens Colletotrichum gloeosporioides and Thielaviopsis paradoxa. [file aem.02575-25-s0004.tif]

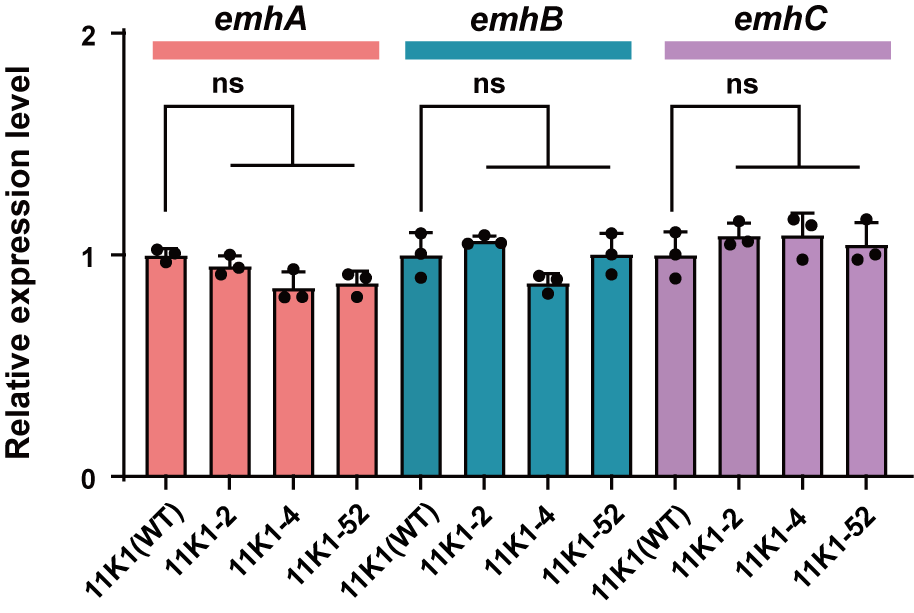

Supplement: Figure S5 — Transcriptional regulation of the emhABC operon by gacA point mutations. [file aem.02575-25-s0005.tif]
