# Supplementary material for: Efflux pump activation confers mupirocin resistance and enhances rhizosphere fitness in Pseudomonas
Source: Appl Environ Microbiol. 2026 Apr 22;92(5):e02575-25. doi: 10.1128/aem.02575-25 (PMC13188873; doi:10.1128/aem.02575-25)
Supplement: Supplemental legends — Legends for Fig. S1 to S5. [file aem.02575-25-s0006.docx]

Supplementary Figure Legends

Supplementary Figure S1 Experimental design of mupirocin MIC assays and biological characteristics of strain 11K1 and its mupirocin-resistant mutants. A. Schematic representation of the mupirocin MIC assay performed via microdilution in LB medium. B–C. Growth curves of 11K1 and mupirocin-resistant mutants in LB medium (B) and M9 minimal medium (C), monitored by measuring OD₆₀₀ values at indicated time points under shaking conditions (at 28°C, 200 rpm). D. Colony morphology of 11K1 and its mupirocin-resistant mutants following single-colony streaking on PDA plates.

Supplementary Figure S2 Specificity of EmhR binding to the promoter p*emhABC*. FAM-labeled EmhABC promoter DNA (FAM-p*emhABC*) was incubated with His₆-EmhR in the presence of increasing concentration of unlabeled p*emhABC*. The binding of FAM-p*emhABC* to EmhR was competitively inhibited by unlabeled p*emhABC* in a concentration-dependent manner, confirming the specificity of the EmhR-DNA interaction.

Supplementary Figure S3 Transcriptional regulation of *emhABC* by EmhR deletion and point mutations. Transcriptional expression levels of *emhA*, *emhB*, and *emhC* in strain 11K1, the Δ*emhR* mutant, and EmhR point mutants (EmhR^A47P^, EmhR^I112T^) at 18 hpi, quantified by qRT-PCR. Statistical significance was determined by one-way ANOVA with Tukey’s post-hoc test: **P < 0.01, ****P < 0.0001.

Supplementary Figure S4 Plate confrontation assays of the Δ*emhR* mutant and EmhR point mutants against the fungal pathogen *Colletotrichum gloeosporioides* (A) and *Thielaviopsis paradoxa* (B). Inhibition zones were observed after 48 h of co-incubation at 28°C.

Supplementary Figure S5. Transcriptional regulation of the *emhABC* operon by *gacA* point mutations. Quantitative RT-PCR analysis of *emhABC* transcript levels in the *gacA* point-mutant strains 11K1-2, 11K1-4, and 11K1-52 (each retaining a wild-type *emhR* allele) at 18 hpi. Statistical significance was determined by one-way ANOVA with Tukey’s post-hoc test: ns, not significant.
