# Supplementary material for: Efflux pump activation confers mupirocin resistance and enhances rhizosphere fitness in Pseudomonas
Source: Appl Environ Microbiol. 2026 Apr 22;92(5):e02575-25. doi: 10.1128/aem.02575-25 (PMC13188873; doi:10.1128/aem.02575-25)
Supplement: Table S1 — Strains and plasmids used in this study. [file aem.02575-25-s0007.docx]

Supplementary Table S1 Strains and plasmids used in this study

| Strain or plasmid | Description | Reference or source |
| --- | --- | --- |
| **Strains** |  |  |
| *P. fluorescens* |  |  |
| 2P24 | wild type; Ap^r^ | Wei and Zhang, 2006 |
| Δ*mup* | *mup* gene in-frame deletion in strain 2P24; Ap^r^ | Lab stock |
| Δ*phlD* | *phlD* gene in-frame deletion in strain 2P24; Ap^r^ | Lab stock |
| Δ*mup*Δ*phlD* | *mup* gene in-frame deletion in strain Δ*phlD*; Apr | Lab stock |
| *P. viciae* |  |  |
| 11K1 | wild type; Ap^r^ | Lab stock |
| Δ*emhR* | *emhR* gene in-frame deletion in strain 11K1; Ap^r^ | This study |
| EmhR^A47P^ | the 47th position of the EmhR protein was changed from alanine to proline in strain 11K1; Ap^r^ | This study |
| EmhR^I112T^ | the 112th position of the EmhR protein was changed from isoleucine to threonine in strain 11K1; Ap^r^ | This study |
| Δ*gacA* | *gacA* gene in-frame deletion in strain 11K1; Ap^r^ | This study |
| Δ*secDF* | *secD* and *secF* genes in-frame deletion in strain 11K1; Ap^r^ | This study |
| SecD^D100E^ | the 100th position of the SecD protein was changed from aspartic acid to glutamic acid in strain 11K1; Ap^r^ | This study |
| SecF^P120S^ | the 120th position of the SecF protein was changed from proline to serine in strain 11K1; Ap^r^ | This study |
| Δ*soxR* | *soxR* gene in-frame deletion in strain 11K1; Ap^r^ | This study |
| SoxR^L147R^ | the 147th position of the SoxR protein was changed from leucine to arginine in strain 11K1; Ap^r^ | This study |
| *P. aeruginosa* |  |  |
| PAO1 | wild type; Ap^r^ | Lab stock |
| Δ*oprM* | *oprM* gene in-frame deletion in strain PAO1; Ap^r^ | This study |
| Δ*nalD* | *nalD* gene in-frame deletion in strain PAO1; Ap^r^ | This study |
| Δ*nalD*/pBBR | Δ*nalD* carrying empty plasmid pBBR; Gm^r^ | This study |
| Δ*nalD*/pNalD | Δ*nalD* carrying recombinant plasmid pNalD; Gm^r^ | This study |
| Δ*nalD*/pNalD^A47P^ | Δ*nalD* carrying recombinant plasmid pNalD^A47P^; Gm^r^ | This study |
| Δ*nalD*/pNalD^I112T^ | Δ*nalD* carrying recombinant plasmid pNalD^I112T^; Gm^r^ | This study |
| *E. coli* |  |  |
| DH5α | DNA cloning and plasmid production. | Lab stock |
| BL21(DE3) | heterologous protein expression. | Lab stock |
| S17-1(λ-pir) | plasmid doner cells that transport recombinant plasmids into P. aeruginosa. |  |
| Fungus |  |  |
| *Thielaviopsis paradoxa* | Plant pathogen that causes Sugarcane stem rot. | Lab stock |
| *Colletotrichum gloeosporioides* | Plant pathogen that causes Mango anthracnose. | Lab stock |
| **Plasmids** |  |  |
| p2P24Km | Suicide plasmid with *sacB* used for homologous recombination; Km^r^ | Li et al., 2018 |
| p2P24-*emhR* | p2P24Km derived plasmid for *emhR* in-frame deletion from strain 11KI; Km^r^ | This study |
| p2P24-*gacA* | p2P24Km derived plasmid for *gacA* in-frame deletion from strain 11KI; Km^r^ | This study |
| p2P24-*soxR* | p2P24Km derived plasmid for *soxR* in-frame deletion from strain 11KI; Km^r^ | This study |
| p2P24-*secDF* | p2P24Km derived plasmid for *secDF* operon in-frame deletion from strain 11KI; Km^r^ | This study |
| p2P24-EmhR^A47P^ | p2P24Km derived plasmid for *emhR* with an amino acid mutant A47P from strain 11KI; Km^r^ | This study |
| p2P24-EmhR^I112T^ | p2P24Km derived plasmid for *emhR* with an amino acid mutant I112T from strain 11KI; Km^r^ | This study |
| p2P24-SecD^D100E^ | p2P24Km derived plasmid for *secD* with an amino acid mutant D100E from strain 11KI; Km^r^ | This study |
| p2P24-SecF^P120S^ | p2P24Km derived plasmid for *secF* with an amino acid mutant P120S from strain 11KI; Km^r^ | This study |
| p2P24-SoxR^L147R^ | p2P24Km derived plasmid for *soxR* with an amino acid mutant L147R from strain 11KI; Km^r^ | This study |
| pBBR1MCS-2 | pBBR1MCS-2 containing pBluescript II KS-lacZ; Km^r^ | Kovach et al., 1995 |
| pBBR1MCS-5 | pBBR1MCS-5 containing pBluescript II KS-lacZ; Gm^r^ | This study |
| pBBR-*iles*^2P24^ | pBBR1MCS-2 containing the *ileS* gene coding region from strain 2P24; Km^r^ | This study |
| pNalD | pBBR1MCS-5 containing the *nalD* gene coding region from strain PAO1; Gm^r^ | This study |
| pNalD^A47P^ | pBBR1MCS-5 containing the *nalD* gene coding region with an amino acid mutant A47P from strain PAO1; Gm^r^ | This study |
| pNalD^I112T^ | pBBR1MCS-5 containing the *nalD* gene coding region with an amino acid mutant I112T from strain PAO1; Gm^r^ | This study |
| pRK2013 | helper plasmid; ColE1 replicon TraRK^+^Mob^+^, Km^r^ | Figurski and Helinski,  1979 |
| pTNS2 | mobilizable helper plasmid encoding only the specific TnsABC+D transposition, *ori*R6K, *ori*T, Ap^r^ | Choi et al., 2005 |
| pCPP6529 | pUC18R6KT-Tn7T-Km, Ap^r^, Km^r^ | Wei et al., 2018 |
| pCPP6351 | pUC18R6KT-Tn7T-Tc, Ap^r^, Tc^r^ | Wei et al., 2018 |
| pEX18 | Suicide plasmid with *sacB* used for homologous recombination; Gm^r^ | This study |
| pEX18-*oprM* | pEX18Gm derived plasmid for *oprM* in-frame deletion.Gm^r^ | This study |
| pEX18-*nalD* | pEX18Gm derived plasmid for *nalD* in-frame deletion.Gm^r^ | This study |
| pET22b | Protein expression plasmid; Ap^r^ | Lab stock |
| pET22b-EmhR | pET22b carrying the *emhR* gene coding region from strain 11KI; Ap^r^ | This study |
| pET22b-EmhR^A47P^ | pET22b carrying the *emhR* gene coding region with an amino acid mutant A47P from strain 11KI ; Ap^r^ | This study |
| pET22b-EmhR^I112T^ | pET22b carrying the *emhR* gene coding region with an amino acid mutant I112T from strain 11KI ; Ap^r^ | This study |

Ap^r^, Km^r^ and Gm^r^ indicate resistance to ampicillin, kanamycin and gentamicin, respectively.

Reference in Table S1

Wei, H. L., Zhang L. Q., 2006. Quorum-sensing system influences root colonization and biological control ability in *Pseudomonas fluorescens* 2P24. Antonie van Leeuwenhoek 89: 267-280.

Li X, Gu GQ, Chen W, Gao LJ, Wu XH, Zhang LQ. 2018. The outer membrane protein OprF and the sigma factor SigX regulate antibiotic production in *Pseudomonas fluorescens* 2P24. Microbiol Res. 206: 159-167.

Kovach, M. E., Elzer, P. H., Hill, D. S., Robertson, G. T., Farris, M. A., Roop, R. M., Peterson, K. M., 1995. Four new derivatives of the broad-host-range cloning vector pBBR1MCS, carrying different antibiotic-resistance cassettes. Gene 166(1), 175–176.

Wei HL, Zhang W, Collmer A. Modular Study of the Type III Effector Repertoire in *Pseudomonas syringae* pv. tomato DC3000 Reveals a Matrix of Effector Interplay in Pathogenesis. Cell Rep. 2018 May 8;23(6):1630-1638. doi: 10.1016/j.celrep.2018.04.037. PMID: 29742421.

Choi KH, Gaynor JB, White KG, Lopez C, Bosio CM, Karkhoff-Schweizer RR, Schweizer HP. A Tn7-based broad-range bacterial cloning and expression system. Nat Methods. 2005 Jun;2(6):443-8. doi: 10.1038/nmeth765. PMID: 15908923.

Figurski DH, Helinski DR. Replication of an origin-containing derivative of plasmid RK2 dependent on a plasmid function provided in trans. Proc Natl Acad Sci U S A. 1979 Apr;76(4):1648-52. doi: 10.1073/pnas.76.4.1648. PMID: 377280; PMCID: PMC383447.
