# Supplementary material for: Efflux pump activation confers mupirocin resistance and enhances rhizosphere fitness in Pseudomonas
Source: Appl Environ Microbiol. 2026 Apr 22;92(5):e02575-25. doi: 10.1128/aem.02575-25 (PMC13188873; doi:10.1128/aem.02575-25)
Supplement: Table S2 — Primers used in this study. [file aem.02575-25-s0008.docx]

Supplementary Table S2 The primers used in this study

| Primers | Sequence (5′→3′) |
| --- | --- |
| *ileS*^2P24^ F | CCGGGGGATCCACTAGTgcagaccgcgactcgcctg |
| *ileS*^2P24^ R | GTGGCGGCCGCTCTAGAgcacaacctgtcgccgccggac |
| *ileS*^11K1^ F | gtcggccaggaagctgaacgct |
| *ileS*^11K1^ R | ctgtatgaccggcgtttgacggtg |
| p2P24 F | GGGTACCGAGCTCGAATTCGTAATCATGGTCAT |
| p2P24 R | GGGGATCCTCTAGAGTCGACCTGCAGG |
| *gacA*-UF | ATTCGAGCTCGGTACCCgctggcgatggggccggagcatg |
| *gacA*-UR | cgcatcactggcatccgacagacctagcgcaaag |
| *gacA*-DF | gtcggatgccagtgatgcgcgcctgttg |
| *gacA*-DR | GACTCTAGAGGATCCCCtgagggaagaagttcttgctgcccaacacc |
| *emhR*-UF | ATTCGAGCTCGGTACCCacagcatcggcccgcaactcaagca |
| *emhR*-UR | cgtggcacgcgtcgtgtcggctgttaccgaccg |
| *emhR*-DF | gacacgacgcgtgccacgcctcgctc |
| *emhR*-DR | GACTCTAGAGGATCCCCtgcctttgaggtcgcgggtcacgcc |
| *secDF*-UF | ATTCGAGCTCGGTACCCtgccagaacttctcccgcgctt |
| *secDF*-UR | gttgctcgcttggtcagacgcaacagaccgcc |
| *secDF*-DF | gtctgaccaagcgagcaactggcgagttcggg |
| *secDF*-DR | GACTCTAGAGGATCCCCagcggattaatccgcgcttctccc |
| *soxR*-UF | TCGAGCTCGGTACCCgtgatattcgtcgaacgacaggcctcg |
| *soxR*-UR | ccagcacatgacggtgagctccttgtggacat |
| *soxR*-DF | caccgtcatgtgctgggcaagcgcg |
| *soxR*-DR | CTCTAGAGGATCCCCaaagccgaccgtggcttttgccg |
| *emhABC*-UF | ATTCGAGCTCGGTACCCaggcggcgtttcttggaatgcg |
| *emhABC*-UR | gatggccgctcctttgaggtcgcgggtcacgc |
| *emhABC*-DF | acctcaaaggagcggccatcagcagctcc |
| *emhABC*-DR | GACTCTAGAGGATCCCCacctttcggtgggcttcttgtttcagcg |
| EmhR^A47P^-UF | ATTCGAGCTCGGTACCCaagctctgacgtcagggtgaagggttg |
| EmhR^A47P^-UR | cagtagatggggccgcgcgtc |
| EmhR^A47P^-DF | cgcgcggccccatctactggcac |
| EmhR^A47P^-DR | GACTCTAGAGGATCCCCgacgcaatcaagacacggcggca |
| EmhR^I112T^-UF | ATTCGAGCTCGGTACCCaggcgcgcaactggcgcactc |
| EmhR^I112T^-UR | ggaacagagtttcattaatgcggcggg |
| EmhR^I112T^-DF | gccgcattaatgaaactctgttccataagtgcg |
| EmhR^I112T^-DR | GACTCTAGAGGATCCCCgtgaatacacctgccggtcagaaatcttcc |
| SecD^D100E^-UF | TTCGAGCTCGGTACCCaggccaagcgcgccaaagag |
| SecD^D100E^-UR | gccacgacgtaatcttcgcccatcg |
| SecD^D100E^-DF | aggcgatgggcgaagattacgtcg |
| SecD^D100E^-DR | CTCTAGAGGATCCCCagcacccggctcagccgcgag |
| SecF^P120S^-UF | TTCGAGCTCGGTACCCttcaccgcgattctcgacgccaac |
| SecF^P120S^-UR | acccacttgcgagccgacga |
| SecF^P120S^-DF | gttcgtcggctcgcaagtgg |
| SecF^P120S^-DR | GACTCTAGAGGATCCCCagccgtgacggatggagcaacgag |
| SoxR^L147R^-UF | TCGAGCTCGGTACCCctcagaaagcccttatggtcccgcg |
| SoxR^L147R^-UR | cctggccccgcagatgcgcgcccg |
| SoxR^L147R^-DF | cgcatctgcggggccaggcgtaagacgttc |
| SoxR^L147R^-DR | CTCTAGAGGATCCCCatgtttcgcgtttgccatccctgt |
| pET22B-F | cccagcctacgcaaaCACCACCACCACCACCACTG |
| pET22B-R | catGGCCATCGCCGGCTGGGC |
| *R6His-F | CCAGCCGGCGATGGCCatggtccgtcgtaccaaagaggaagc |
| *R6His-R | GGTGGTGGTGGTGGTGtttgcgtaggctggggctcaggc |
| p*emhABC*-F | GAAATCCTCGGGTCCAGGCGCG |
| p*emhABC*-R | CACCTCAATCAGGATGCGTAAGCAATGCG |
| pEX18Gm-F | ctagaggatccccgggtaccgagc |
| pMD18Gm-R | agtcgacctgcaggcatgcaagc |
| PAO1*oprM*-UF | cccggggatcctctagtccatcaccatcgtgtcggccatg |
| PAO1*oprM*-UR | cacgccgcggcaccatctcgcccttgtc |
| PAO1*oprM*-DF | tggtgccgcggcgtggacggtagcgg |
| PAO1*oprM*-DR | catgcctgcaggtcgactgtgaagatcggcaggttgatcgacgg |
| PAO1*nalD*-UF | cccggggatcctctagaggcgtgtcagggtcagcagg |
| PAO1*nalD*-UR | agaccgatgagccgatgttccgtggcct |
| PAO1*nalD*-DF | catcggctcatcggtctcggcaacgggg |
| PAO1*nalD*-DR | tgcctgcaggtcgactggctcagctggacgtgctcga |
| pBBRGm-F | ACTAGTGGATCCCCCGGGCTGCAGGAATTC |
| pBBRGm-R | TCTAGAGCGGCCGCCACCGCGGTGGAGCTC |
| CNalD-UF | gggggatccactagttcggtggcaggcaaggcacttc |
| CNalD-DR | tggcggccgctctagaacgtccaggtggatcttggcgc |
| CNalD^A47P^-UR | tagacggggccacgggtcaccccg |
| CNalD^A47P^-DF | cccgtggccccgtctactggcacttccag |
| CNalD^I112T^-UR | gcatcagggtggtcaggatgcggcgcttctt |
| CNalD^I112T^-DF | ctgaccaccctgatgcaacgttgcgaattc |
| qP*emhA*-F | gcggtagtagtcctggttggcg |
| qP*emhA*-R | ccgcgttccaggaagtctccg |
| qP*emhB*-F | ggcacccagaagatcgccaggatg |
| qP*emhB*-R | gtggtcccgctggcgatttcc |
| qP*emhC*-F | ccgtcttccagggtcagcttgacc |
| qP*emhC*-R | gtcgacgtgacgcaaagctcg |

* indicates that the nucleotide sequences of the 6-His-tagged EmhR, EmhR^A47P^, and EmhR^I112T^ proteins were all amplified by PCR using this pair of primers.
